# Coffee Ring Effect Enhanced Surface Plasmon Resonance Imaging Biosensor via 2-λ Fitting Detection Method

**DOI:** 10.3390/bios14040195

**Published:** 2024-04-16

**Authors:** Youjun Zeng, Dongyun Kai, Zhenxiao Niu, Zhaogang Nie, Yuye Wang, Yonghong Shao, Lin Ma, Fangteng Zhang, Guanyu Liu, Jiajie Chen

**Affiliations:** 1 School of Physics & Optoelectronic Engineering, Guangdong University of Technology, Guangzhou 510006, China; 2112115091@mail2.gdut.edu.cn (D.K.); 17793175617@163.com (Z.N.); zgnie@gdut.edu.cn (Z.N.); malin@gdut.edu.cn (L.M.); zhang.ft@gdut.edu.cn (F.Z.); liuguanyulh@163.com (G.L.); 2 School of Physical Science and Information Technology, Liaocheng University, Liaocheng 252059, China; 3 Key Laboratory of Optoelectronic Devices and Systems of Ministry of Education and Guangdong Province, College of Physics and Optoelectronic Engineering, Shenzhen University, Shenzhen 518060, China; yuyewang@szu.edu.cn (Y.W.); shaoyh@szu.edu.cn (Y.S.)

**Keywords:** surface plasmon resonance, coffee ring effect, biosensing and bioimaging, biophotonics and plasmonics

## Abstract

SPR biosensors have been extensively used for investigating protein–protein interactions. However, in conventional surface plasmon resonance (SPR) biosensors, detection is limited by the Brownian-motion-governed diffusion process of sample molecules in the sensor chip, which makes it challenging to detect biomolecule interactions at ultra-low concentrations. Here, we propose a highly sensitive SPR imaging biosensor which exploits the coffee ring effect (CRE) for in situ enrichment of molecules on the sensing surface. In addition, we designed a wavelength modulation system utilizing two LEDs to reduce the system cost and enhance the detection speed. Furthermore, a detection limit of 213 fM is achieved, which amounts to an approximately 365 times improvement compared to traditional SPR biosensors. With further development, we believe that this SPR imaging system with high sensitivity, less sample consumption, and faster detection speed can be readily applied to ultra-low-concentration molecular detection and interaction analysis.

## 1. Introduction

Proteins are essential components of cells and tissues, playing a vital role in maintaining cell growth and renewal, information exchange, and the normal immune function of the body [1]. The study of protein–protein interactions is a crucial tool for exploring life science issues, particularly in immunological research, where analyzing the binding process of antigens and antibodies can provide critical information for disease diagnosis, drug development, and disease treatment mechanisms [2,3]. To date, many immunological detection methods have been proposed and applied, such as enzyme-linked immunosorbent assays (ELISA) [4], immunofluorescence [5], chemiluminescence [6], and surface plasmon resonance (SPR) [7]. Among these technologies, SPR biosensors, which provide unique real-time and lab-free detection capabilities, have become an important tool for exploring the kinetics of biomolecular interactions [1,8,9].

The refractive index resolution (RIR) is a critical factor in the performance of surface plasmon resonance (SPR) biosensors. The higher the RIR, the lower the detection limit of the biosensors. In SPR biosensors, a signal response will be generated when the target biomolecules bind with the biological probe on the surface of a metal film. However, through Brownian motion, the probability of the target molecules coming into contact with the surface of the sensor chip is greatly limited, resulting in the system being able to detect fewer molecules than the total number of molecules actually contained in the liquid to be tested. In SPR systems used to study molecular interactions, a micro flow cell structure is commonly employed to continuously circulate the solution being tested. This flow creates a certain degree of disturbance in the solution, which increases the molecular contact probability. 

By conducting molecular enrichment, the local molecular concentration can be increased, and so too the probability of molecular contact with the sensor chip, thus attaining biosensors with high resolution. Nowadays, SPR sensing technology has achieved high-resolution detection of biomolecules by combining various methods of enhancement. Several enhancing methods have been developed in passive and active ways [10]. The passive modes include SPR-enhancing materials, sensing substrates, or signal amplification tags [11,12]. The active modes usually employ an external light field or electric field to drive the molecular enrichment, such as electrokinetic transportation, plasmonic optical, and optothermal methods [13,14]. Compared with the passive modes, the active enrichment mode can effectively solve the fundamental problem for solution-based reactions; the interaction efficiency between biomolecules is diffusion limited. This is particularly problematic when testing low-concentration analytes, as it can be time- and sample-consuming. For example, our group has proposed an all-optical active method termed optothermophoretic flipping (OTF) [15], which is the first temporal modulated method for protein–protein interaction enhancement. However, the optothermal method requires additional heating lasers, and the heat generated by these lasers can limit their wider bio-applications. Here, we adopted the coffee ring effect (CRE) for local molecular enrichment via open droplet microfluidics. The CRE is generated during the evaporation process of the droplet, where particulate matter inside the droplet moves continuously to the edge of the droplet, resulting in the sample being enriched by continuously gathering at the edge [16]. It is also used in combination with biosensing technology to detect the biomarkers of body fluids, such as blood, saliva, and tears, etc. [17,18,19]. The CRE-based SPR detection method is not only heating-free; it also has several advantages over enclosed microfluidic flow cells, such as lower sample consumption, simpler operation, and lower cost, due to the use of open droplet microfluidics [20]. 

The SPR imaging (SPRi) technique allows for the monitoring of any region of interest on the sensing surface and for the identification of the biomolecule enrichment area, making it suitable for combining with the CRE method to achieve detection at lower concentrations. Typically, there are four interrogation modes of SPRi sensing: intensity, angle, phase, and wavelength. Among them [21], the wavelength interrogation SPRi interrogation mode (WSPRi) can freely select the optimal excitation light wavelength for different samples and has a large dynamic range for covering the refractive index (RI) difference of the various samples to be tested [22,23,24]. Therefore, WSPRi sensing is more suitable for imaging detection. Furthermore, by means of enrichment, the molecular binding efficiency can be effectively improved, which requires high-time-resolution performance from the sensor to monitor the reaction process. One method of achieving this is by scanning the wavelength of the incident light and using the 2D detector to detect the reflected light [25]. When one scanning cycle is completed, the SPR spectrum of all sites on the whole sensing surface can be obtained, effectively improving the time resolution. However, such systems inevitably require the use of wavelength scanning devices, including acoustic optic tunable filters (AOTF) [26,27], liquidcrystal tunable filters (LCTF) [28,29], or monochromators [30], which can be expensive and have response times ranging from 4 ms to 1 s. Although many algorithms have been proposed in previous research to improve the time resolution by reducing the number of scanning points required for each scanning cycle, the inherent response time of the spectroscopic device limits the time resolution of WSPRi sensing technology, leaving room for further improvement.

In this paper, we propose a novel WSPRi-biosensors-based 2-λ fitting algorithm, by which we can calculate the entire SPR spectrum using only two data points in the spectrum. The system employed two cost-effective light sources with filters of specific wavelengths to scan the incident wavelengths, eliminating the need for expensive spectroscopic devices and greatly improving the time resolution. Additionally, we combine the CRE to increase the local molecular concentration and the probability of molecules contacting the chip surface probe, thus improving the RIR of WSPRi. Furthermore, without the use of an enclosed microfluidic cell, our droplet-based scheme offers several advantages over traditional microfluidic cell-based methods, including passive biomolecular enrichment, reduced sample consumption, and faster detection speed. Our proposed WSPRi biosensor presents a significant advancement in SPR-based sensing technology and offers the merits of high sensitivity, cost-effectiveness, and ease of use for a wider range of biomedical applications.

## 2. The Principle of the 2-λ Fitting SPRi Detection Method

Theoretically, the SPR spectral curve can be expressed by Formula (1); the $R_{0}$, A, D, W, and $\lambda_{0}$ refer, respectively, to the source intensity, asymmetry, depth, width, and RW (resonance wavelength) [30,31]. Among them, three parameters can be readily confirmed and fixed before detection: the parameter $R_{0}$ can be obtained by directly detecting the incident light, and the parameters A and D barely change during the detection process and can be acquired from the spectral curve with a spectrometer before the experiment. Thus, there are only two unknowns in the Formula (1), and the RW can be calculated from two data in the spectrum. First, we use the Fresnel formula to generate the ideal SPR spectrum as a real signal [29]; then, we generate a set of reference spectra via Formula (1). In Formula (1), three parameters—A, D, and R_0_—are determined, and two parameters—$\lambda_{0}$ and W—are used as variables. By changing parameters $\lambda_{0}$ and W, we can obtain a series of reference signals: $I_{\lambda 1}^{\prime }$ and $I_{\lambda 2}^{\prime }$. In order to improve the SNR (signal–noise ratio), two main factors for SPR excitation wavelengths λ_1_ and λ_2_ need to be considered: (1) the wavelength used for calculation should be far away from the RW band with weak reflectivity; (2) the corresponding incident light intensity should be increased. When using incoherent broadband light sources (such as a halogen lamp LED, etc.), it is common to have strong light intensity in the visible light band; therefore, selecting this region as the excitation band can improve the signal-to-noise ratio of the measurement. Based on the above analysis, we employed LEDs as an excitation light source and chose 600 nm and 690 nm for calculating the RW.

(1) $$R\left(\lambda \right)=R_{0}\times \left(1-D\frac{W+A(\lambda -\lambda_{0})}{W^{2}+{\left(\lambda -\lambda_{0}\right)}^{2}}\right)$$

**Figure 1:**
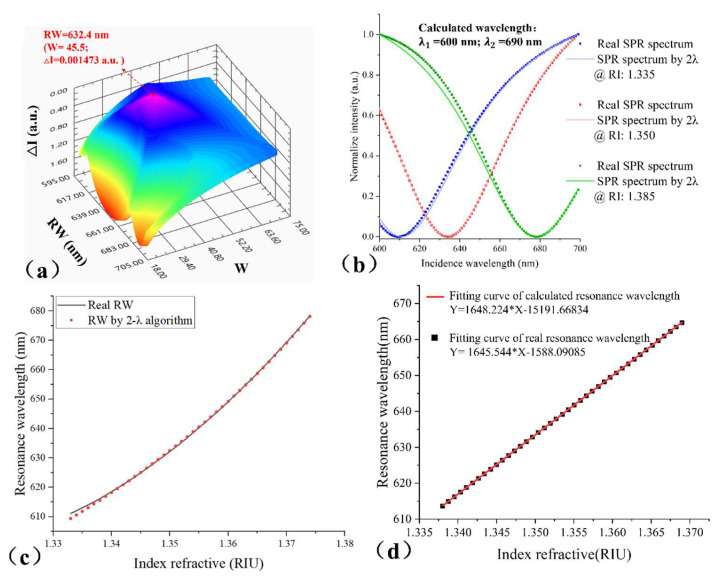
The 2−λ fitting method description and error analysis: (**a**) the 3D plot of the change of ∆I with the W and RW; ∆I = 0.001473 a.u. is the minimum value when the RW = 632.4 nm and W = 45.5; (**b**) the SPR spectral curve via the 2−λ algorithm and real spectrum with different RI; (**c**) the RW via the 2−λ algorithm and the real RW with RI; (**d**) comparison of linear fitting between the RW via the 2−λ algorithm and the real RW in the applicable RW range.

After obtaining the reference signal, we explain the data processing process in Figure 1a. The difference between the reference signal and the actual signal ($I_{\lambda 1}$ and $I_{\lambda 2}$) is acquired: $∆I=\left|I_{\lambda 1}^{\prime }-I_{\lambda 1}\right|+\left|I_{\lambda 2}^{\prime }-I_{\lambda 2}\right|$. When the ∆I is minimum, the $\lambda_{0}$ is considered to by the actual RW. The real SPR spectrum and the spectrum calculated via 2-λ algorithms are shown in the Figure 1b; the red curve shows the good accuracy of the algorithm, but the green and blue spectra have some errors. The reason for the errors is that the parameters A and D are fixed, and the two parameters have a slight deviation in the spectral change process. So, we analyze the application scope of the algorithm. Figure 1c presents the 2-λ-algorithm-obtained RW and real RW versus RI curve. From the results, the RI (refractivity index) went from 1.338 to 1.369 RIU, and the error of RW is in the order of 0.01 nm. The error within this range is of the same order of magnitude as the calculation accuracy, so the 2-λ algorithm is applicable within this range. We analyzed the accuracy of the 2-λ algorithm within the scope of application; Figure 1d shows the relationship between RI and 2-λ-algorithm-obtained and real RW separately via linear fitting. The slope of both curves is almost consistent, and the error is about 0.18%. Thus, the applicable RW range of the 2-λ algorithm is 623.5 nm to 676.03 nm, corresponding to the RI range in the order of $10^{-2}$ RIU. 

## 3. Optical Setup

**Figure 2:**
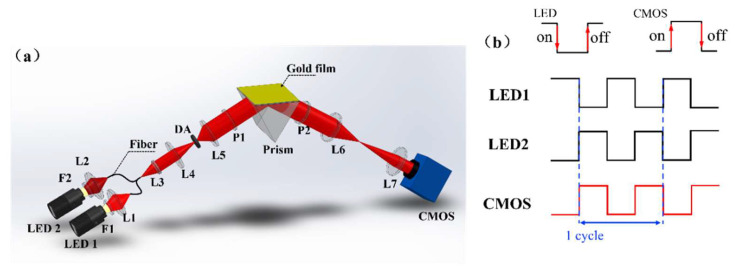
The WSPRi system: (**a**) schematic diagram of system optical path (L1–7: lens; P1–2: polarizer; DA: diaphragm aperture); (**b**) working sequence diagram of LED and camera in the system.

The optical schematic of our optical setup is shown in Figure 2a. The system is designed in three parts: incidence arm, sensing module, and reflect arm. In the incidence arm, two LEDs are used as the SPR excitation light source, and two filters are used to acquire the monochromatic light; then, the two lights are coupled into the Y-type fiber via two objective lenses. The central wavelengths of F1 (FB620-10, Throlabs, New York, NY, USA) and F2 (FB690-10, Throlabs, New York, NY, USA) are 600 nm and 690 nm, respectively, and the full width at half height (FWHM) values of the two filters are both 10 nm. The light from the fiber is collimated by the lens group and incident to the sensing module. The sensing module consists of a prism (equilateral triangle with side length of 18 mm made by SF11, 1.785 RIU) and a sensor chip. The gold film in the system is based on 1 mm-thick glass (length: 18 mm; width: 18 mm) as the substrate, with a 48 nm-thick gold layer deposited on the substrate through evaporation, and 2 nm of chromium layer spacing between the glass substrate and the gold coating to increase the fixation effect. In this module, the incident light is coupled through a prism, and the SPR phenomenon is excited at the interface between the metal and the sample. Finally, the reflected light passes through the imaging lens and is acquired by CMOS (DMK 23GV024, Imaging Source, Bremen, Germany). The system is controlled by home-made Labview software (2019, 32-bit). When the system starts working, the two LEDs (LB-L21-64-WDG, power: 5 W; Lubang Photonics Technology, Changsha, China) blink alternately, and the sensing surface is imaged by the CMOS. On the sensing imaging surface obtained by the CMOS, each pixel can serve as an independent sensing unit, so we can extract the SPR signal at any position on the sensing surface. This performance is conducive to the comparative study of the enrichment effect of droplets in different regions under the CRE.

Figure 2b shows the system’s working sequence diagram. When LED 1 is on, the CMOS images the sensing surface once; then, LED 1 is off, LED 2 is on, and the CMOS images the sensing surface again, which forms a scanning cycle. Every time a scanning cycle is completed, the RW of all sites on the sensing surface will be obtained through the 2-λ fitting algorithm. 

## 4. Results and Discussion

### 4.1. Chemicals and Materials

The goat anti-human igG, human igG, and phosphate-buffered saline (PBS) were obtained from Solarbio Science and Technology Co., Ltd. (Beijing, China). The anti-mouse IgG (H + L) was offered by Proteintech group, Inc. (Chicago, IL, USA). Refractive index matching oil (RI = 1.780) was bought from Cargille Laboratories, Inc. (Cedar Grove, NJ, USA). NaCl was obtained from Aladdin (Shanghai, China).

### 4.2. RIR and Dynamic Range of WSPRi Measurement

**Figure 3:**
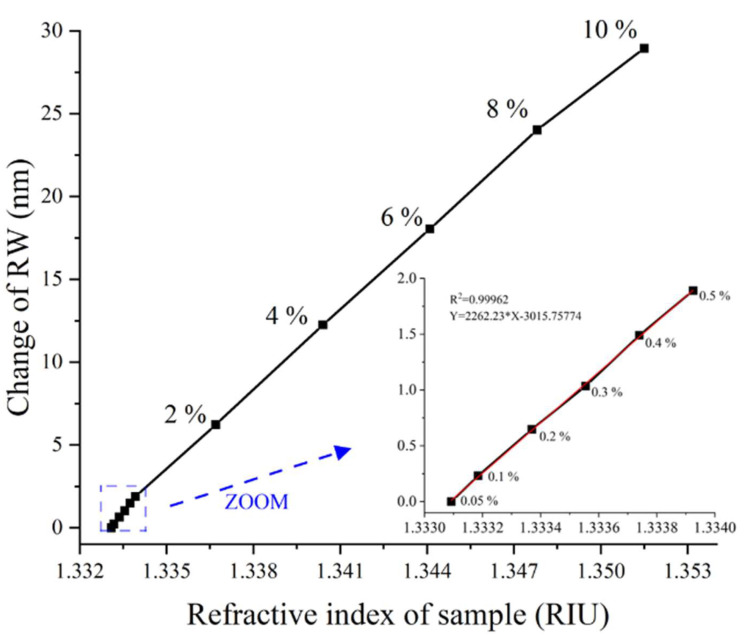
The RW varies with different concentrations of saline; the inset fig. is the change in the RW caused by low-concentration NaCl solution.

The RIR and dynamic range are important parameters for biosensors. Here, we tested the performance of the system by monitoring a NaCl solution with different concentrations. In the experiment, we set the incidence angle at 54.3°, which is the optimal incident angle for 0.05% NaCl solution according to the Fresnel formula. A NaCl solution with low concentration levels from 0.05% to 0.5% was detected to calculate the RIR of our system, as shown in the zoomed-in view in Figure 3. Each NaCl solution was measured three times; for all concentrations, the maximum deviation of RW was less than 1.2 nm. According to Formula (2), the RIR of our system is calculated as $\sigma_{RI}=1.76\times 10^{-6}$ RIU, where $\sigma_{n}$ = 0.0008325 RIU is the change in RI, $\sigma_{\lambda }=1.8899$ nm is the corresponding RW response, and ${\Delta \sigma }_{SD}=0.004$ RIU is the root mean square of noise. We performed linear fitting on the data, and the linear correlation coefficient ($R^{2}$) of the data was 0.9962, which exhibits a nearly linear relationship between the shift in RW and the change in RI.

(2) $$\sigma_{RI}=\frac{\sigma_{n}}{\sigma_{\lambda }}{\Delta \sigma }_{SD}$$

Then, we also tested NaCl solutions with concentrations ranging from 2% to 10% in increments of 2% by volume. The corresponding RW response is shown in Figure 3. The results show linear correlations between the RW and RI of the sample (concentration ranging from 0.05% to 10%; RI ranging from 1.3330925 RIU to 1.3515000 RIU). Therefore, the dynamic range of the system is 0–0.0184075 RIU and reaches the order of 10^−2^ RIU [29]. The maximum amplitude rate of the CMOS is 4.5 frame/ms, and the delay time of the LED is 0.006 ms. In each scanning cycle of the system, the LED and CMOS work twice, respectively. Therefore, the fastest imaging speed can be calculated as $t_{i}=2\times \left(4.5\;ms+0.006\;ms\right)=9.012\;ms$.

### 4.3. CRE SPR Enhancement

**Figure 4:**
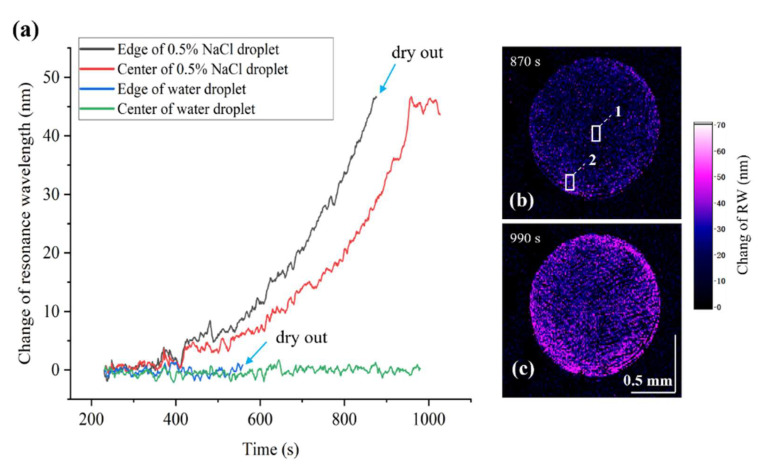
Dynamic monitoring of the near-field ion concentration distribution during the evaporation of the droplet: (**a**) real-time response curves for center and edge positions of brine and pure water during evaporation; (**b**,**c**) the change in RW images by false color at different times.

To verify the real-time image monitoring performance of the system for the formation process of the CRE, we monitored the evaporation process of saline droplets. The temperature and humidity of the experimental environment are 20° and 42%, respectively. In the experiment, we drop the pure water (1.5 μL) and NaCl solution (0.5% concentration; 0.5 μL) and monitor the evaporation process through our system. We detect the real-time RW at different sites, as demonstrated in Figure 4a. For the NaCl droplet, the RWs of the center and edge positions (shown in the small frame of Figure 4b) are exhibited by the black and red curves in Figure 4a. It can be clearly observed that the RW change in the droplet edge position is faster than that in the center. This phenomenon can be explained by the enrichment of the sodium chloride at the edge of the liquid caused by the CRE. Since no molecules or particles are present in the pure water, no aggregation will take place in the evaporation process. As a result, the RI of both the edge and the center region in the water droplet remains unchanged, as depicted by the green and blue curves. Figure 4b,c present the RW image of the NaCl droplet at different times. Figure 4b presents the RW imaging during the evaporation process of the salt solution. In Figure 4c, the NaCl concentration in the edge position has saturated, while the center area has not yet reached the saturation state, which indicates a faster RW change at the edge and is in good accordance with the results in Figure 4a.

### 4.4. Antibody-Antigen Binding Enhancement with CRE

The local concentration of biomolecules can be largely increased via molecule enrichment induced by the CRE. The probability of the target molecules binding with the probes on the sensing surface will also be increased, which improves the RIR of biosensors. In this section, we monitored the reaction process of goat anti-human IgG and human IgG to prove the biosensing performance of the system. The target solution consists of both PBS and goat anti-human IgG. In the evaporation process, PBS will also have an enrichment effect and cause RW signal changes via the CRE. In addition, the evaporation efficiency of PBS with different volumes will vary. To eliminate the interference of PBS on the test results and determine the optimal volume, we first monitored the evaporation process of pure PBS with different volumes.

**Figure 5:**
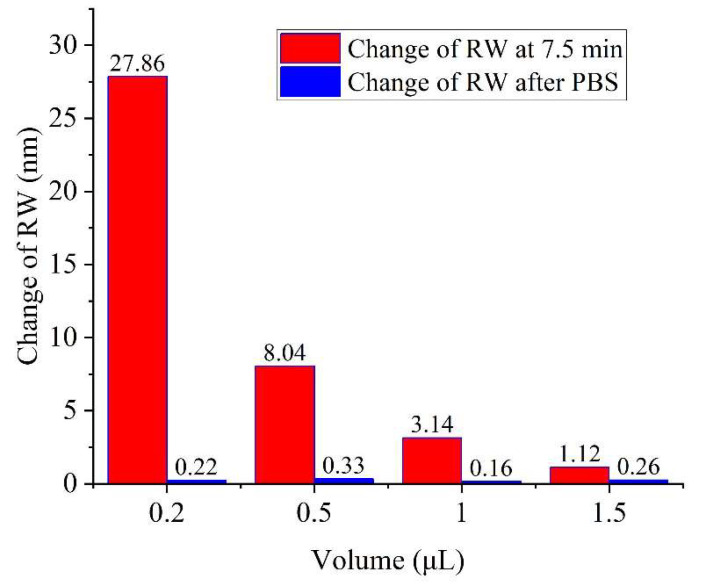
Detection of RW at 7.5 min and after PBS drop.

We dropped the PBS solution at different volumes (0.2, 0.5, 1, and 1.5 μL) on the gold film surface; when the edge is about to be dried (at approximately 7.5 min), excessive PBS is dropped. We extracted the RW signal at the edge position of the droplet (CRE-driven molecular enrichment region), and the result is shown in Figure 5. The experimental results show that the RW signal can return to the baseline position before evaporation after excessive PBS is dropped (as shown in the blue bar chart in Figure 5). Moreover, we recorded the SPR signal of the edge region (enrichment area under the CRE) of the droplet with different volumes at about 7.5 min. The resulting RW signal changes of 27.86, 8.04, 3.14, and 1.12 nm corresponding to volumes of 0.2, 0.5, 1 and 1.5 μL, respectively, as shown by red bar chart in Figure 5. According to the above results, droplets with smaller volume have better enrichment effects under the CRE, which enhances the sensing performance and reduces the usage of reagents as well. However, limited by the imaging resolution of the system, the sample volume cannot be reduced infinitely. In order to better observe the signals at each site of the droplet, we choose 0.2 μL as the experimental volume condition.

Then, we monitored the process of antigen–antibody-specific binding under the CRE. In this process, the CRE was adopted to enrich the protein molecules to be tested and to lower the detection limit. Before the experiment, the surface of the sensor chip is modified through the following three steps: (1) cleaning—the sensor chip is washed with PBS (0.01 M, pH = 7.3); (2) fixing—the chip is immersed in the human IgG (100 μg/mL) solution and shaken with a shaking table for 15 min, then washed by PBS; (3) blocking—the chip is immersed in the BSA (bovine serum albumin) solution with the concentration of 100 μg/mL to block the non-specific binding sites on the chip, followed by another PBS wash.

**Figure 6:**
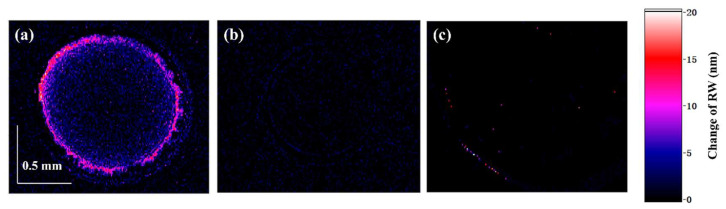
RW imaging results under different conditions: (**a**) goat-anti-human IgG with 500 ng/mL liquid to be tested; (**b**) the liquid to be tested is PBS; (**c**) anti-mouse IgG (H + L) with 500 ng/mL liquid to be tested.

After modifying the sensor chip, we dropped goat anti-human IgG with 0.2 μL onto the surface of the chip. After about 6 min, a PBS with 0.5 mL was dropped for dispersing the non-specific molecule. In the excess PBS solution, the bound antibody remains unaffected on the surface of the sensor chip, and the error caused by non-specific adsorption is reduced. The reaction time of the antigen and antibody is usually about 5 min [18,32], while the edge of 0.2 μL PBS is dried up for about 7.5 min, which is enough to cover the binding process of the antigen and antibody. The change in RW imaging before and after the reaction is shown in Figure 6a. In order to verify whether the RW difference is caused by antigen–antibody binding, we conducted a control experiment. Instead of dropping goat anti-human IgG solutions, we dropped PBS into the sensor chip modified with IgG probe and monitored the RW imaging results 6 min later. As shown in Figure 6b, no significant RW difference before and after the reaction was observed. As a result, we can assert that the difference in RW in Figure 6a is indeed caused by biomolecule binding. To verify the influence of non-detection target proteins in the test solution, we conducted comparative experiments. The temperature, humidity, and processing of the sensing chip were all consistent in the experiment. During the experiment, we dropped anti-mouse IgG (H + L) (concentration: 500 ng/mL; volume: 0.2 μL) onto the surface of the sensing chip (with human igG as the probe molecules) and let it sit for 7.5 min before adding 1 mL of PBS solution. The experimental results, as shown in Figure 6c, showed that due to the inability of anti-mouse IgG to bind specifically to human antibodies on the chip surface, there was no significant change in RW after the reaction. Therefore, the impact of non-target proteins in the test solution on detection was minimal.

**Figure 7:**
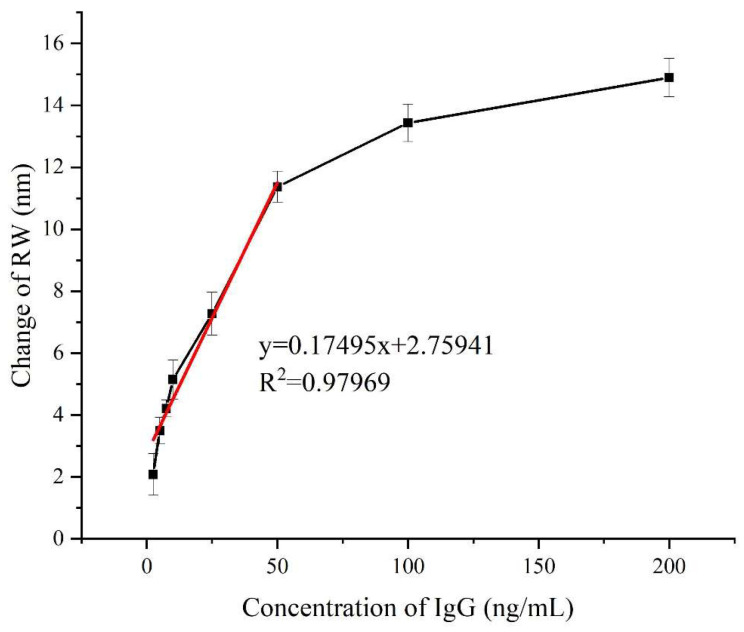
Calibration plot corresponding to the shift in RW with respect to IgG concentration, the black curve represents the RW signal corresponding to different concentrations of IgG (2.5, 5, 7.5, 10, 20, 50, 100, and 200 ng/mL), and the red curve represents a linear fitting curve within the range of 2.5 ng/mL to 50 ng/mL.

Furthermore, we detected goat anti-human IgG with different concentration levels (2.5, 5, 7.5, 10, 20, 50, 100, and 200 ng/mL), and each experiment is repeated three times, as demonstrated in Figure 7. From the results, which exhibits a nearly linear relationship between the shift of RW and the protein concentrations ranging from 2.5 to 50 ng/mL. Compared to WSPRi based on the micro flow cell without enhancement, the CRE method has the following advantages: (1) the signal is enhanced by about 383.5 times [15]; (2) simple operation without any additional liquid circuit device; (3) less reagent consumption; only a 0.2 μL sample is needed to complete the test. In addition, the limit of detection (LOD) of our platform was calculated according to the following equation [33]:

(3) $$LOD=\frac{C}{R}\times \sigma_{SD},$$

where C is the lowest concentration of the analyte, R is the sensor response according to the lowest concentration of analyte binding with its ligand, and $\sigma_{SD}$ is the RMS noise of our platform. For our platform, these three parameters are 2.5 ng/mL, 2.08 nm, and 0.04 nm, respectively. Therefore, the LOD of our platform is 0.0340 ng/mL (213 fM).

## 5. Conclusions

We developed a simple and highly sensitive WSPRi system that differs from conventional WSPRi by using two LEDs combined with specific filters instead of expensive light splitters. We also proposed a 2-λ fitting algorithm to solve the reflectivity width (RW) problem. Our results showed that the RIR and dynamic range are $1.76\times 10^{-6}$ RIU and 0–0.0184075 RIU, respectively. Additionally, the CRE was used to induce biomolecular enrichment. It has the advantages of less sample consumption and simple operation. The sensing performance of WSPRi has been effectively enhanced without applying an additional thermal field, electric field, or magnetic field. Nevertheless, there is still room for the optimization of this WSPRi sensing device. First, the spatial resolution can be improved by using the aspherical imaging lens group, which makes the RW signals of each point on the sensing surface more accurate; second, a highly sensitive 2D detector can be used to suppress dark noise for higher sensitivity; finally, the sensitivity can be further improved by using filters with better performance to obtain monochromatic light with a narrower bandwidth as the incident light. For the biosensor based on CRE enhancement, the temperature, humidity, chip substrate materials, and volume of liquid drops to be measured in the experimental environment can also be further optimized. In summary, we have introduced a novel SPRi biosensor enhanced by the CRE, which boasts a range of advantages such as cost-effectiveness, high sensitivity, and real-time capabilities. Especially noteworthy is its capability of detecting and analyzing biomolecular interactions even at ultra-low concentrations, signifying its importance in the field of biotechnology and biosensing.

## Data Availability

No new data were created or analyzed in this study. Data sharing does not apply to this article. We used only publicly available datasets for experimentation.
